# Supplementary material for: Coping strategies of Ghanaian couples after unsuccessful infertility treatment: An exploratory qualitative study
Source: PLoS One. 2025 Jun 27;20(6):e0326923. doi: 10.1371/journal.pone.0326923 (PMC12204582; doi:10.1371/journal.pone.0326923)
Supplement: S1 File — (DOCX) [file pone.0326923.s001.docx]

**Interview Transcription on Coping Strategies**

**Client 1**

**Emotional Coping Strategies**

*How do you cope with the emotional challenges of infertility?*

I tried seeking treatment at some point in my youth when the problem started. I was not working I was not working so I didn’t have enough money to cater for the bills. But about year ago I started experiencing bloated stomach so I decided to go to hospital to see a doctor. The doctor told me to do a scan, and it was identified that I had some fibroid that needs to be removed. But because of my age, the doctor advised that I try to conceive before I do the surgery. I am 45 years now, but I never thought I was getting to menopause that will make childbearing permanently impossible. I was surprised to know that if I don’t take steps now, that might be the end of my dream of having a child in my lifetime.

Since I tried in my youth and it was not coming, I decided to take my mind of it. It has been too long since this problem persisted. I was not interested staying with any man in order to forget about it. You don’t feel like socializing with peers where they discuss issues related to children. Motivation to do everything is gone. I check myself if there is something wrong with me that is not making me conceive. We have done all the investigations but there is nothing wrong with any of us. When I was not working, I used to think about it a lot, but when I got a job and it was paying well, the trauma of always thinking about it decreased.

**Coping with Uncertainty**

*How do you manage the uncertainty and unpredictability of infertility so far?*

Is all about God. God is my only source of hope. To man it looks impossible but with God all things are possible. I know one day I will also smile because God is with us. It is a very difficult situation and it can even make you feel sick all the time. Sometimes we feel empty, as if we are not part of human beings. If not the fact that this problem (fibroid) I am experiencing is renewing my interest in childbirth, I would have closed my mind.

Both family and friends are expecting much from us. That is how our society is, the expectation is too huge, and we are very worried too. It is not all family gathering that we are able to attend because of this problem. But there is nothing much that we can do on our own, we rely on the almighty for his help. I attend church, I pray to God everyday about the situation but nothing happens. I am not getting what I want.

**Decision-Making and Coping with Setbacks**

*Can you describe a difficult decision you've had to make regarding infertility?*

Everything is difficult. Yah, everything. Is kind of affects everything including planning for the future. There is too much uncertainty. There is nothing much I can say, God is our only helper.

**Client 2**

**Emotional Coping Strategies**

*How do you cope with the emotional challenges of infertility?*

When I stayed with my partner for over two years and nothing was coming, I really got shocked about the problem. Where from this, why me? I asked myself several times. Ei God, don’t let the worse happen to me. It has not been easy. Sometimes I will sit down and think ‘saa’. Every month ‘biaa’ when my menstruation comes, I feel bad, although it doesn’t affect my job or my work. But when I am alone in the house, I start to think about it. I do not have any specific coping strategy; I only be myself as much as possible. I don’t involve myself in other people’s issues in order not to be hurt by anyone.

**Coping with Uncertainty**

*How do you manage the uncertainty and unpredictability of infertility so far?*

We should have married a long time but because of this problem that is why it is keeping it long. The man wants to see evidence before he goes ahead to perform the marital rights. Now my marriage is in limbo because of my inability to conceive. You know in our part of the world; all eyes are on the woman to conceive. I always have to get closer to my parents in order to become okay. My parents and my siblings are my source of joy whenever I see them.

*Have you experienced any feelings of inadequacy or guilt as a result of infertility? How do you cope with those feelings?*

I was staying with one guy before I met my current guy. I did three abortions with the guy. So I don’t know why. Sometimes, I ask whether my fault or the fault of the guy. But the guy too is having one child already, so I don’t know why. I don’t know if is because of the abortion that is causing this problem. I go to church, but I don’t blame God for my situation. I feel guilty for myself rather than blaming God. But I pray that the Lord will favor me in these trying moments.

**Decision-Making and Coping with Setbacks**

*Can you describe a difficult decision you've had to make regarding infertility?*

The uncertainty about the future makes it difficult to plan anything. I have had to absent myself from a lot of social gatherings, both at church and school because anytime I see children in social gathering, it reminds me of my plight and I begin to cry. That notwithstanding, I still entertain children and embrace them. I do try to organize things on my own and do what is best for myself. When faced with difficult tasks I sit down with my partner sometimes and plan things with him on the way forward. We need to also talk to our doctors on the next line of action because you can’t just ignore it and be okay.

**Client 3**

**Emotional coping strategies**

*How do you cope with the emotional challenges of infertility?*

I have had this problem for five years now. The family keeps giving pressure. That really hurts me. My mum will call and say it is keeping long oo, be hurry and give birth to the second one. I had no major worries since I already had one child. I used not to think about it so much. We started planning for a second child when she was between 3-4 years. But now that it is not coming, we are getting worried about it. I always pray to God for his mercy upon my life so that I can have another child. My pastor has also been praying with us for God’s grace to conceive.

*Can you describe a time when you felt particularly overwhelmed by the emotions associated with infertility? How did you manage those feelings?*

We have done series of test but they don’t find any problem. And yet we could not conceive for over five years. That was really shocking. I was like how do we get solution to the problem if there is nothing wrong with us. They have put me on clomid and M2 tone before. Yet the problem is not resolved. That is very challenging. But we only take consolation from the child we have already.

**Coping with Uncertainty about treatment outcome**

*How do you manage the uncertainty of infertility so far?*

This problem can make me lose focus sometimes because the society expects much from you and your partner. I feel demotivated and confused. I feel like I have no value, always questioning myself, what is actually wrong. I only trust in the almighty to help me find solution to this problem one day in my life. Because to man it is impossible, looking at how our tests found no problem, and yet, we could not conceive again. The doctors are doing their part but God is the final say. We will not give up but continue to pursue treatment and count on God for a miracle.

**Decision-Making and Coping with Setbacks**

*Can you describe a difficult decision you've had to make concerning the outcome of your infertility treatment? How do you cope with setbacks?*

I stay with my husband and we have one child who is 8 years old. I have had miscarriage before, when the pregnancy was about 6 months. We have tried since then but no results. It is not a good experience. Sometimes you don’t feel like making love with your partner. But my partner is always available and keeps assuring me that it is not over yet.

Client 4

**Emotional coping strategies**

*How do you cope with the emotional challenges of infertility?*

I have been going through a lot since I started experiencing this problem. Sometimes I don’t even know what to do. I become emotionally down and unable to focus on certain things. I try to relax and sometimes find a rest by sleeping if I am at home. I also pray to God to help me deal with this situation. He is the source of my hope. Sometimes too, I only withdraw from situations that make me remember my plight. If the problem is internal, ie from my partner or a close relative, I try to distance myself and avoid arguing with them.

I remember, one time I met colleagues in a gathering, young ones like me and each of them was carrying their own child. You can imagine. It is not a pleasant situation at all. Is like when they are talking anything about children and challenges involved in raising them, I sort of become dumb. I ask myself several questions as why this is happening to me. I then pretended I had a call, left the scene for some time to cry at the washroom, until I was able to put myself together again and returned to my seat. It is very unpleasant situation, but what can I do, just praying to God for help.

**Coping with uncertainty about treatment outcome**

*How do you manage the uncertainty of infertility so far?*

It looks very frustrating and unpleasant. It is not a situation I would wish for anyone. When you think about it, is like you don’t know what to do. I ask myself: is that the end of it all for me? Sometimes I give up, although I believe that it is not all over yet since this is just the beginning of treatment. I listen to some people’s success story and I say to myself, it is all not over yet.

**Self-Identity and Self-Esteem**

*How has infertility affected your sense of self-worth and identity?*

Hmmm. I don’t even know what to say. Sometimes I feel like I am empty. As if there is nothing good about me. People look down upon you just because I cannot give birth. People can gossip about you as if you are not a human being. Our society is such that if you are not able to give birth, you are not regarded as any important person, or someone who can reason and that usually puts me off.

*Have you experienced any feelings of inadequacy or guilt as a result of unsuccessful infertility treatment? How do you cope with those feelings?*

Guilt is a big problem for me when I begin to think about the problem sill persisting after going through a series of investigations and treatment. I ask myself if I have done something bad or is a curse on me. I don’t know why I am experiencing this problem. All my friends who have married have their kids and I am still struggling. But my partner has been supportive all this while. He consoles me anytime I look depressed because of this problem and I tend to forget about it

**Decision-Making and Coping with Setbacks**

*Can you describe a difficult decision you've had to make concerning the outcome of your infertility treatment? How do you cope with setbacks?*

Sometimes, I feel like selling all that I have, not to think of the food I will eat, just to look for solution to this problem. Facing this challenge is like you can’t hear anything again. We are looking forward to even selling our properties to engage in advance treatment if we don t get positive results within the next few years because age is weighing on us. I try to discuss the problem with my mother whenever encounter new challenges or setbacks. I am so close to my mum, and she sometimes offer me the help and encouragement that I need in any difficult situation.

Client 5

**Emotional coping strategies**

*How do you cope with the emotional challenges of infertility?*

Anytime I think of the problem I am going through; my heart becomes disturbed. I tend to lose focus on whatever I am doing. What can I do? Is like it has taken over all my concentration. I sometimes engage in a conversation with a close friend just to take my mind of some stuff. I do video calls with closed family relations, watch television and sleep. Aside that, nothing interests me anymore.

*Can you describe a time when you felt particularly overwhelmed by the emotions associated with infertility? How did you manage those feelings?*

I have had several occasions that I have encountered uncomfortable scenes. I went for a funeral in my hometown one day and people will be asking you questions as if I am the one to put baby in my stomach. Some of them can even tell you to your face; ‘madam, be hurry and get pregnant oo because age is catching up on you’. Whiles I am doing my best to find solution to the problem, others talk as if the matter does not concern me. Is very pathetic but what can I do. I leave everything to God.

**Coping with Uncertainty about treatment outcome**

*How do you manage the uncertainty of infertility so far?*

I cannot do much about the outcome. The doctors know best and I believe they will do their humanly possible best to get me the results I need. They are those I count on now. I will not give up yet. I only pray for more of Gods strength and count on him for a better outcome. With God all things are possible.

**Self-Identity and Self-Esteem**

*How has infertility affected your sense of self-worth and identity?*

I am always worried, sometimes without knowing what to do. I feel like I am not part of human beings. Why having this challenge that I never thought of is very sad. Planning my life become difficult because the future looks uncertain. That is very said.

*Have you experienced any feelings of guilt as a result of infertility? How do you cope with those feelings?*

Hmmm, I don’t know whether to blame myself or anyone for this menace. This problem has reduced my confidence in everything I do. But I know from within my heart that I have not done anything to bring about this problem. I don’t know whether it is my fate or what. But in all things, I rely on the Almighty for help.

**Decision-Making and Coping with Setbacks**

*Can you describe a difficult decision you've had to make concerning the outcome of your infertility treatment? How do you cope with setbacks?*

This situation makes planning of my life very difficult. You don’t know whether all that we are toiling for is going to who. The major worry is that we are aging but cannot still decide on properly plan our life. I remember one time how it became very difficult for me to use my siblings instead of my own children as beneficiaries for my end of service benefit. I asked myself ooh Lord so when. I was hoping to get the expected results but it didn’t go that way. That is not easy to deal with, but since that is not the end of it all, I only assure myself that I will conceive one day

Client 6

**Emotional Coping Strategies**:

*How do you cope with the emotional challenges of infertility?*

The emotional trauma is not a good experience at all. You think about it and you begin to panic. When I am alone in the house, I get so disturbed. Although I am a first-degree holder, I decided to further my education by doing my masters so that I can always have people around me. When I am with people, the emotional distress goes down. That is the reason why I engaged in the master’s program just to keep myself busy and get associated with friends all the time

*Can you describe a time when you felt particularly overwhelmed by the emotions associated with infertility? How did you manage those feelings?*

So many times, is uncountable. The feelings were very bad. Once at a social gathering when they called parents to come and stand by their children. It looked like I was the only odd one out. I almost cried. Very unpleasant feelings. Hmmm, I don’t wish anyone to go through that experience. When it happens like that, you only have to leave the scene and console yourself. That’s all, but it was really sad one indeed.

**Coping with Uncertainty about treatment outcome**

*How do you manage the uncertainty of infertility so far?*

If you decide to always think about when you are going to get outcome, you will always feel sick. So, the best thing is to take your mind off for some time and focus on what you are doing.

**Self-Identity and Self-Esteem**

*How has infertility affected your sense of self-worth and identity?*

Hmmm, you know everyone’s wish is to get their own children. Living as a couple without children makes you look like you’re empty, not really good is seen around you. The only thing is that I don’t lose hope irrespective of how people look at us.

*Have you experienced any feelings of inadequacy or guilt as a result of infertility? How do you cope with those feelings?*

I have no feelings of guilt concerning this problem. Its nature that determines the fate of everyone. I know all things work together for good.

**Decision-Making and Coping with Setbacks**

*Can you describe a difficult decision you've had to make concerning the outcome of your infertility treatment? How do you cope with setbacks?*

Planning for the future is very difficult for us. How do you plan without children, when you are not sure when about when you are going to get one? But we believe that all hope is not lost since we have more years ahead of us. Once God is alive, we still have faith that one day we will see changes.

**Client 7**

**Emotional Coping Strategies**:

*How do you cope with the emotional challenges of infertility?*

The pain, agony and distress, hmmm, is not everything that I can say now. Sometimes you don’t feel like talking to anyone at all. You only think about when you are going to get your own child. Imagine going to church every Sunday, you see people in Sunday school class, and yet, you have tried your best and not getting it. You begin to ask God why so long time? But in all these I have learnt one thing and that is never give up

*Can you describe a time when you felt particularly overwhelmed by the emotions associated with infertility? How did you manage those feelings?*

So many times, my heart gets broken. It seems crying is the only solution to decrease these anxieties and worries. I can’t count the number of times I have cried because of this problem. I remember one time, I corrected a child who was misbehaving in our neighborhood. I did not even beat her, only verbal warning. But she reported the issue to her parents, and the mother came to attack me, casted insinuations, by saying; do you know how to go through birth pains. I was really sad. My husband consoled me and that was it. But I could not even eat the whole day because of that.

**Coping with Uncertainty about treatment outcome**

*How do you manage the uncertainty of infertility so far?*

It is very frustrating that you can’t even know who is going to inherit all that we are toiling for. We don’t even know the kind of treatment we should go for again. It is money involving too. Now the next option is to may be look for money and seek advanced support like IVF. It is not easy; we only have to press on without given up. May God have mercy on us.

**Self-Identity and Self-Esteem**

*How has infertility affected your sense of self-worth and identity?*

Where I come from, if you are not able to give birth, the stigma is not easy. High value is placed on children. Is like you are not respected when you are not able to reproduce. Sometimes I feel shy going to our hometown because you go there and children as low as 14 years have given birth, and marrying for almost 5 years, no child. The people look at you in some way.

*Have you experienced any feelings of inadequacy or guilt as a result of infertility? How do you cope with those feelings?*

Hmmm, as for guilt, I do not even know what to say. I know that this problem is not because of my own fault no my partner. Only God knows why. We know it is not over yet, we still hope for the best.

**Decision-Making and Coping with Setbacks**

*Can you describe a difficult decision you've had to make concerning the outcome of your infertility treatment? How do you cope with setbacks?*

We can’t even plan anything money about the future. Almost all the money we are earn goes into treatment. Our building project is stalled now because of this problem.

Client 8

**Emotional Coping Strategies**:

*How do you cope with the emotional challenges of infertility?*

I have gone through a lot of emotional traumas since I started experiencing signs of infertility. The pain, and worry are not easy, hmmm. Both societal and family pressure keeps mounting on us to give birth. Friends are expecting that you conceive, church members, as well as family members. At times, when they ask you about it, they may sound like they are reassuring you, but that keeps reminding me you the emotional distress. I only reassure myself and call friends to converse with.

**Coping with Uncertainty about treatment outcome**

*How do you manage the uncertainty of infertility so far?*

I am not sure if our marriage is even going to hold forever. I can see that sometimes the man is almost losing interest in me. He accompanies me to clinic, all tests are done, but there is nothing wrong found. I suspect he may even think that I am the cause of the problem. My marriage is even not secured without children. That is how I see it. The man may go behind me and take another woman. I only pray for Gods strength. When I am down emotionally because of these thoughts, I call my parents and they reassure me.

**Decision-Making and Coping with Setbacks.**

*Can you describe a difficult decision you've had to make concerning the outcome of your infertility treatment? How do you cope with setbacks?*

Almost everything becomes difficult. Unless you say that you don’t care about it. And I don’t think anyone experiencing this problem will say that they don’t care about it. At a point in time, I feel like quitting church because I don’t even know how to pray again. I just hope that my next available treatment will bring me positive results. We have trust in the professionals and I know they are doing their best to help us. We will continue to seek their advice on the next alternative option

Client 9

**Emotional Coping Strategies**:

*How do you cope with the emotional challenges of infertility?*

The situation is not easy to cope with. Sometimes, when I am teaching in school, I become depressed. Especially, when colleague talking about children, I see disappointment in my life. To come to this world without children, it is better not to be born at all. I don’t really have any specific thing to rely on as a means of coping. I only count on God to save the situation for us.

**Coping with Uncertainty about treatment outcome**

*How do you manage the uncertainty of infertility so far?*

Sometimes all hope is lost. You see nothing good about the future. I even ask myself sometimes; should I end it all. My first partner deserted me, found another woman and they conceived. So, I am not sure if the problem is from me. I try as much as possible to erase it from my thoughts and everything that I am doing. It is not all that easy but that is what helps me to get going with other business for survival.

**Decision-Making and Coping with Setbacks**

*Can you describe a difficult decision you've had to make regarding infertility?*

I have faced the difficulty of deciding whether to get someone to stay with but I am afraid to embark on that decision because I think that might take our attention away from seeking further treatment. We are just allowing ourselves some time to see the treatment outcome before certain issues can be dealt with conclusively.

Client 10

**Emotional Coping Strategies**

*How do you cope with the emotional challenges of infertility?*

I am always worried and distressed. Anytime I become sad about the situation, I call my mother. She has been reassuring me. She encourages me all the time and that gives me hope. Sometimes my parents even suggest some places to go for treatment and support me financially too. As you know, infertility treatment is very expensive. Myself and my partner’s finances are not even enough to cater for all the costs of treatment. My parents have been providing financial support for me and my partner all this while.

**Coping with Uncertainty**

*How do you manage the uncertainty and unpredictability of infertility so far?*

My hope is that, one day I will wake up to see a positive pregnancy test. For now, although what we are looking for is not yet achieved, we will keep praying while we seek care from professional sources. Also, I try to remain focused on what I am doing because if i decide to think about it all the time, I cannot even do my work well, yah. So that is fine with me

**Decision-Making and Coping with Setbacks**

*Can you describe a difficult decision you've had to make and how you deal with setbacks?*

We needed to sell some cloths and other properties to get money for this treatment. Now the outcome shows that, we are not through yet and we need to save more money for further treatment. These are the challenges we are facing. Hmmm. Is draining us financially but what can we do. We only need to press on to get what we want.

**Client 11**

**Emotional Coping Strategies**:

*How do you cope with the emotional challenges of infertility?*

As for coping, I can’t think of any suitable strategy. Is not something that you can easily develop antidote to it and say you have forgotten about it. But in all these, isolation from certain places decreases the anxiety.

Sometimes you hear people given testimony on television and you’re like ei, what is happening to me? At one time, the place that someone was testifying about, I have been there several times but I could not conceive. That made me panic because it makes me think that my problem is probably worse. I had to build my faith and reassure myself that Gods time is the best

**Coping with Uncertainty**

*How do you manage the uncertainty and unpredictability of infertility so far?*

Seeking treatment without results is very uncomfortable. Not getting the results means you have to reassure yourself. No one can reassure you better than doing it yourself. For we can’t tell when

**Decision-Making and Coping with Setbacks**

*Can you describe a difficult decision you've had to make regarding infertility?*

I do not have any difficulty regarding decision making. I go about my duties as usual, just like any other individual. I always motivate myself.

Client 12

**Emotional Coping Strategies**:

*How do you cope with the emotional challenges of infertility?*

It has not been easy all this while. Stress associated with it is not easy. It makes me lose focus sometimes. But always reassure myself that, once we are alive, there is still hope for the future. When we could not achieve conception after following all the doctor’s instructions, we were very sad. I kept asking myself; so when? Hmmm, is not easy. But my hope is always in God

**Coping with Uncertainty**

*How do you manage the uncertainty and unpredictability of infertility so far?*

I reassure myself that once we are seeking professional help, we will get the results we need. The doctors are doing all that they could, and we hope for the best. Of course we cannot do that without prayers, so we pray to God for his help too.

**Decision-Making and Coping with Setbacks**

*Can you describe a difficult decision you've had to make regarding infertility?*

We became devastated and could not concentrate on anything for like 2 weeks. After spending all this money on investigations and treatment and still not seeing the results. It is not easy. But what can we do, only God knows our fate, and determines the future ahead of us.

Client 13 (Male)

**Emotional Coping Strategies**:

*How do you cope with the emotional challenges of infertility?*

The emotional pain associated with it, if I decide to think about it all the time, I will not be able to go about my normal duties. Anytime I become saddened by the situation, I try to maintain my composure as a man. I do that to make sure the woman does not become deeply worried. Sometimes I leave home to engage myself in social activities such as watching football with friends. At other times too, I engage in hiking or cycling just to keep myself busy so I won’t continue thinking about the problem.

Although at times, and in some gatherings, some friends may intentionally pass comments that depicts that if I can’t work on the woman, somebody will take her from me, all because she is not getting pregnant, but I just ignore them, and sometimes leave the scene to avoid further emotional agony. Such comments sadden me a lot sometimes, although they may think that they are joking, it can really hurt me. Of course, they are my friends so what can I do. I just pray to God that this problem will not last forever.

**Coping with Uncertainty**

*How do you manage the uncertainty and unpredictability of infertility so far?*

It has not been an easy experience so far. The emotional discomfort is not easy. Sometimes we try to suppress it. This is not the first time we have sought treatment; we were hoping for positive outcome but it has not been the case so far.

**Decision-Making and Coping with Setbacks**

*Can you describe a difficult decision you've had to make regarding infertility?*

Hmmm, as for difficult decisions, it is usually recurring. How to properly plan for the future becomes difficult. Like you think of what you are going to do with all the struggles if I cannot reproduce. Sometimes I decide to forget about everything.

**Client 14 (male)**

**Emotional Coping Strategies**:

*How do you cope with the emotional challenges of infertility?*

Prayer is my motivation. I have the strongest hope in God that I will surely hold my baby one day. God will not disappoint us. I also engage in social activities and church activities. I keep myself busy just to forget about it because the more you think about it, the more you become sad.

My heart bleeds anytime I see children perform at church or in school. I ask myself so how long I’m I going to wait to see results. Hmmm. We just take assurance from our parents. Sometimes they may sound like putting pressure on us, but when they see that we are getting too emotional, they tend to counsel us.

**Coping with Uncertainty**

*How do you manage the uncertainty and unpredictability of infertility so far?*

You only need to take your mind of it and that is all. If you decide to think about it every day, you may even die early.

**Self-Identity and Self-Esteem**

*How has infertility affected your sense of self-worth and identity?*

There is high level of stigma attached to it and that makes it uncomfortable experience. It brings about sadness and emotional instability. You can even lose focus on what you are doing, and it happen to me most often.

**Decision-Making and Coping with Setbacks**

*Can you describe a difficult decision you've had to make regarding infertility?*

Nothing seems to matter anymore. You can’t even think about yourself, likewise resolving a difficult situation. All attention is only on finding solution to the problem. You hear so many adverts and places, you get confused about where to start from. All the herbals and so forth. I don’t want to be easily moved by these adverts although is not easy to do that. I only try as much as possible to rely on what health professionals are doing for me, and hope for a better outcome until they say something different.

**Client 15 (Male)**

**Emotional Coping Strategies**:

*How do you cope with the emotional challenges of infertility?*

I mostly spend time with friends just to keep my company so that I will not always become bored. As a man, even though I become emotionally down due to this challenge, I try as much as possible to suppress it in order not to make the woman so sad as well. You know they are weaker vessels and turn to we the men for support.

When we finished the treatment and treatment and there was no sign of conception, my spirit almost left me. It wasn’t easy. My wife almost cried when she saw blood (menses) on two occasions following treatment.

**Coping with Uncertainty**

*How do you manage the uncertainty and unpredictability of infertility so far?*

I share my free moments with family and friends. I do not want to become bored since that will remind me of the pain. I want to build my carrier in furthering my education, just to keep me busy all the time. I also take consolation from the fact that it is not over yet. Even if we need to go for assisted reproduction, we will do so. We can’t give up at this stage

**Decision-Making and Coping with Setbacks**

*Can you describe a difficult decision you've had to make regarding infertility?*

At a point in time, it became very difficult for me whether I should forget about seeking additional treatment. We have tried our best, so many places, from herbal, prayer camps and so on. But we still trust God

Client 16

**Emotional Coping Strategies**:

*How do you cope with the emotional challenges of infertility?*

I keep myself busy in my profession. I always associate myself with friend so I do not become bored. I show love to children and consider them as my own whenever the need arises. People may be asking about what the actual problem is and what you are doing about it. Others suggest various forms of therapy, including local herbals. Sometimes, if you don’t take care, you will be tossed between here and there. But in all these we try to remain focused and pray as well.

**Coping with Uncertainty**

*How do you manage the uncertainty and unpredictability of infertility so far?*

Hmm. It is very difficult to tell what is ahead of you in the future, hence the only thing is to leave everything in the hands of the Lord.

**Decision-Making and Coping with Setbacks**

*Can you describe a difficult decision you've had to make regarding infertility? How do you cope with setbacks?*

When it comes to decision taking and problems with setbacks concerning the problem, especially about seeking treatment and where, and the outcome, I discuss with my partner. We usually have no challenge about were and how to seek treatment. only that whenever I suggest things like local herbals, my partner usually disagrees and that can hurt a bit. Knowing the kind of testimonies people give concerning such treatments. But in all these, I seek advice from my mother on what to do next, especially when previous treatment didn’t go well as expected.

Client 17

**Emotional Coping Strategies**:

*How do you cope with the emotional challenges of infertility?*

It has not been easy all this while. I just hope in God that everything will be okay one day. Although it was not easy when my wife tested positive for pregnancy and she later had a miscarriage. But thanks for all the help and encouragement. As we are speaking now, there are goosebumps all over my body. That positive pregnancy even came when we were least expecting anything like that. Although it didn’t stay due to miscarriage, is gives us assurance that she will definitely conceive again. Now the doctor says she should have enough rest so we want go by his advice.

**Coping with Uncertainty**

*How do you manage the uncertainty and unpredictability of infertility so far?*

There is nothing too had for God to do. We are seeking treatment, and we will keep praying too. We understand the challenge is not easy to overcome but we will not lose hope. It only requires patience and endurance.

**Decision-Making and Coping with Setbacks**

*Can you describe a difficult decision you've had to make regarding infertility? How do you cope with setbacks?*

We have had to take a very difficult decision due to the problem. I had to let my wife stop her job. This is due to the advice we had from the doctor. Because she conceived and it got miscarriage, the doctor advised that she rests for some time. I then decided to let her stop her cooking job and stay home for some time. As we are speaking now, she is not returning to her job anytime soon. I will let her remain at home. Although it is not going to be easy financially, we need to remain focused and let some things go.

Client 18

**Emotional Coping Strategies**:

*How do you cope with the emotional challenges of infertility?*

As for the emotional dilemma, it is not easy to deal with. Comments from friends and some colleagues can make things worse. You only have to ignore it from your thoughts and pray your prayer. If you decide to give attention to all that you see and hear, it can even compound the problem.

**Coping with Uncertainty**

*How do you manage the uncertainty and unpredictability of infertility so far?*

The situation makes it difficult to cope but we try our best so that it doesn’t our plans. Making some decisions is not easy. You ask yourself a lot of questions as to how to plan certain things. We tend to give priority to the treatment, that’s what we spend most of our money on. We believe the early we tackle the issue the better. We just hope and believe that things will work out faster. I know we will surely get what we are looking for one day.

**Decision-Making and Coping with Setbacks**

*Can you describe a difficult decision you've had to make regarding infertility? How do you cope with setbacks?*

We have had to keep fasting and praying. It requires a lot of sacrifice and dedication. People see us sometimes and they think we don’t have money to buy food and eat just because of fasting. We also seek family support too. They also offer a lot of advice and motivation. Our pastor too has been praying for us. I also try as much as possible to concentrate on my job and not to focus on only the problem
